# Affective Neural Responses Modulated by Serotonin Transporter Genotype in Clinical Anxiety and Depression

**DOI:** 10.1371/journal.pone.0115820

**Published:** 2015-02-12

**Authors:** Desmond J. Oathes, Lori M. Hilt, Jack B. Nitschke

**Affiliations:** 1 Department of Psychiatry and Behavioral Sciences, Stanford University, Stanford, CA, United States of America; 2 Department of Psychology, Lawrence University, Appleton, WI, United States of America; 3 Departments of Psychiatry and Psychology, University of Wisconsin-Madison, Madison, WI, United States of America; University of Pennsylvania, UNITED STATES

## Abstract

Serotonin transporter gene variants are known to interact with stressful life experiences to increase chances of developing affective symptoms, and these same variants have been shown to influence amygdala reactivity to affective stimuli in non-psychiatric populations. The impact of these gene variants on affective neurocircuitry in anxiety and mood disorders has been studied less extensively. Utilizing a triallelic assay (5-HTTLPR and rs25531) to assess genetic variation linked with altered serotonin signaling, this fMRI study investigated genetic influences on amygdala and anterior insula activity in 50 generalized anxiety disorder patients, 26 of whom also met DSM-IV criteria for social anxiety disorder and/or major depressive disorder, and 39 healthy comparison subjects. A Group x Genotype interaction was observed for both the amygdala and anterior insula in a paradigm designed to elicit responses in these brain areas during the anticipation of and response to aversive pictures. Patients who are S/L_G_ carriers showed less activity than their L_A_/L_A_ counterparts in both regions and less activity than S/L_G_ healthy comparison subjects in the amygdala. Moreover, patients with greater insula responses reported higher levels of intolerance of uncertainty, an association that was particularly pronounced for patients with two L_A_ alleles. A genotype effect was not established in healthy controls. These findings link the serotonin transporter gene to affective circuitry findings in anxiety and depression psychopathology and further suggest that its impact on patients may be different from effects typically observed in healthy populations.

## Introduction

Conceptualization of psychopathology is at a crossroads, garnering heightened interest among researchers and clinicians alike. The substantive dialogue of the past decade regarding the classification of mental illness contributed importantly to revisions for DSM-5 [1,2]. Also receiving consideration were alternatives to symptom-based approaches of classification, giving rise to the Research Domains Criteria (RDoC) initiative [3], which challenges a number of conventions in psychiatric research. This initiative proposes moving beyond traditional diagnostic categories to conduct research on a range of psychopathology with similar features, such as anxiety and depressive disorders. Another emphasis of the RDoC initiative is on biological measures. Although research to date did not warrant greater inclusion of biological criteria in DSM-5, gains have been made in identifying promising brain and genetic contributions to anxiety and depressive pathology. The field of neuroimaging genetics is uniquely positioned to inform ongoing developments regarding psychiatric diagnosis.

The serotonin system has been strongly implicated in affective psychopathology and associated brain areas, as indicated by research at the non-human animal level [4] and by genetic association studies and neuroimaging studies in humans and non-human primates [5]. Medications that affect serotonin levels in the brain by inhibiting the reuptake of serotonin into the pre-synaptic terminal are first-line treatments for clinical anxiety and depression. Serotonin levels are also affected by the serotonin transporter gene, *SLC6A4*, on chromosome 17q11.1-q12, which encodes 5-HTT protein. The transcriptional activity of this gene is modulated by a variable number of tandem repeats for the linked polymorphic region (5-HTTLPR), most commonly occurring as 14 repeats for the short (S) allele or as 16 repeats for the long (L) allele. A second variant in the 5-HTT promoter region, an A to G single nucleotide polymorphism (rs25531), greatly reduces mRNA expression in the L_G_ compared to L_A_ carriers, rendering the L_G_ variant functionally similar to the S allele [6]. S/L_G_ allele carriers compared with L_A_ homozygotes have largely reduced serotonin transporter expression, fewer 5-HT_1A_ receptors, and increased adrenocorticotropic hormone responses to stress [7]. However, the consequences for serotonin reuptake and extracellular serotonin levels remain controversial [8,9].

In numerous studies of non-psychiatric groups, which have only rarely used the triallelic classification, S carriers have shown greater amygdala activity to emotional stimuli than individuals with two L alleles, although the effect size is small [10]. The few triallelic studies with patient samples thus far show mixed results [10,11,12,13]. Early notions that the S allele promote anxiety have not always been supported, with amygdala responses often normative in S carriers and diminished in individuals with two L alleles [14,15]. Regardless, greater amygdala activation in S/L_G_ carriers than L_A_ homozygotes is consistent with studies reporting 5-HTTLPR differences in attention biases to threat, fear-potentiated startle, and neuroendocrine responsivity [5,16] which predict exaggerated reactivity for S/L_G_ carriers in our experimental context.

The critical role of the serotonin system in affective disorders makes the need for research in patient samples paramount. Patient studies that also assess 5-HTTLPR genetic effects are obscured by methodological complications such as: including medicated patients (mostly with serotonergic drugs) [13,17,18], not including healthy comparison subjects [17,18,19], and not including the triallelic genotype classification [18,19]. A notable exception investigating unmedicated MDD patients using the triallelic assay found evidence of low gene expression associated with greater amygdala activation to aversive pictures in depressed patients compared with controls [12]. Expanding on this work in a related sample, the present study addresses the impact of triallelic 5-HTTLPR genotype on affective neurocircuitry in unmedicated anxious patients across the related and commonly comorbid conditions of generalized anxiety disorder (GAD), social anxiety disorder (SAD), and major depressive disorder (MDD). GAD is characterized by chronic worry that is difficult to control about a number of different topics. MDD diagnosis includes consistent depressed mood and/or diminished interest or pleasure in normal activities and an SAD diagnosis reflects clinical levels of anxiety concerning social and evaluative situations. Clinically, all three disorders typically present with symptoms in common such as negative affect/distress, sleep problems, and concentration problems. Anticipation and response to emotional information are expected to be discontinuous with healthy participant data in patients and are assessed here using our task designed to activate neural circuits in healthy controls [20,21,22] as well as patients [23] including those brain regions of paramount interest to the present investigation, the amygdala and anterior insular cortex, as explained below.

The amygdala and insula are both broadly implicated in social-emotional processing [24,25], in the anticipation of aversive events [26], and in group effects associated with affective disorders compared with healthy populations [24]. There is a strong non-human animal literature on the role of the amygdala in fear learning and memory [27] which is thought to parallel development of affective disorders in humans [28,29,30]. The anterior portion of the insular cortex is particularly linked to theoretical work on anxiety [20,31,32], especially as it involves sensory information [25] with the integration of salient or emotional material [33,34,35].

Hyperreponsivity of the amygdala and anterior insula are the two consistent functional findings for SAD [24]. Those same two areas are among the most frequently showing effects in GAD [23,36,37], as well. A recent meta-analysis found that the amygdala and anterior insula are also implicated in MDD pathophysiology [38]. Some studies in all three disorders have failed to find evidence of amygdala and/or insula hyperactivity [23,39,40,41,42], which may be partially explained by within-group differences including 5HTTLPR genotype. The present study expands the investigation across these disorders using an anticipatory anxiety paradigm designed to specifically tap brain circuitry associated with anxiety and depression and previously shown to activate the amygdala and anterior insula [20,21,22,43]. The notion of anticipation is essential to describe anxious states and is fundamental to descriptions of anxious pathology [44]. This study tested whether variations in affective neural responses found in prior studies of GAD, SAD, and MDD are influenced by 5-HTTLPR genotype across those diagnostic categories. We also sought to extend findings from diverse behavioral and biological assessments of 5-HTTLPR genotype in healthy participants to investigations of the anticipation of emotional information, given our previous fMRI findings in healthy and patient populations [20,21,22,23,45].

## Materials and Methods

### Participants

The study was authorized by the University of Wisconsin institutional review board and after complete description of the study at the outset of the first experimental session, all participants gave written informed consent before participating and no minors were included in the research. The authors assert that all procedures contributing to this work comply with the ethical standards of the relevant national and institutional committees on human experimentation and with the Helsinki Declaration of 1975, as revised in 2008. All 89 participants were administered the Structured Clinical Interview for DSM-IV by DJO and postdoctoral/graduate—level clinicians, with diagnostic discrepancies resolved by consensus of those clinicians and JBN after viewing videotaped interviews. Healthy comparison subjects (*n* = 39) had no current or past neurological or psychiatric disorders and reported no family history of mental illness. Patients (*n* = 50) included 24 who met criteria for GAD but no other current Axis I disorder (15 of those also had no other past Axis I diagnosis, 7 with past MDD), 9 who met criteria for GAD and depression but not SAD (7 MDD, 1 dysthymia, 1 MDD and dysthymia), 7 who met criteria for GAD and SAD but not depression (2 with past MDD, 1 with past MDD and dysthymia), and 10 who met criteria for all three. The only other observed diagnoses in the patient group were for substance use (9 past substance dependence, 1 current substance abuse, 1 past alcohol abuse). Participants were excluded if they reported a history of psychosis or bipolar disorder. No participant was taking psychiatric medications during the study (for patients with medication history, time since last use exceeded 3 half-lives). Table 1 lists demographic and genotyping information, and Table 2 provides scores on the Hamilton Rating Scales of Anxiety (Ham-A) [46] and Depression (Ham-D) [47], the Penn State Worry Questionnaire (PSWQ) [48], and the Intolerance of Uncertainty Scale (IUS) [49].

**Table 1:** Demographic and genotypic information for patients and healthy comparison subjects.

	Healthy Comparison Subjects (*n* = 39)	Patients (*n* = 50)
Age, Mean (SD)	24.64 (6.88)	27.38 (10.07)
Sex, *n* (%)	21 female (53.8)	38 female (76.0)
Ethnic Background, *n*		
Europe	30	41
Africa	2	3
Far East Asia	6	5
Undeclared	1	1
5-HTTLPR Genotype, *n* (%)		
L_A_L_A_	9 (23)	14 (28)
L_A_L_G_	2 (5)	3 (6)
SL_A_	20 (51)	21 (42)
SL_G_	1 (3)	3 (6)
SS	7 (18)	9 (18)
5-HTTLPR Genotype Grouping, *n* (%)		
S/L_G_ carriers (L_A_L_G_, SL_A_, SS, SL_G_)	30 (77)	36 (72)
L_A_ homozygotes (L_A_L_A_)	9 (23)	14 (28)

**Table 2:** Symptom data for 5-HTTLPR groupings in patients and healthy comparison subjects.

5-HTTLPR Grouping	Ham-A	Ham-D	PSWQ	IUS
Healthy Comparison Subjects (*n* = 39), Mean (SD)				
S/L_G_	1.51 (1.33)	2.22 (1.59)	33.53 (8.13)	60.27 (16.65)
L_A_L_A_	1.06 (0.84)	1.58 (1.57)	29.56 (7.60)	52.63 (10.10)
Patients (*n* = 50), Mean (SD)				
S/L_G_	17.55 (6.47)	26.06 (8.83)	61.71 (9.79)	105.14 (24.12)
L_A_L_A_	17.32 (6.14)	27.24 (11.29)	58.43 (10.35)	114.29 (22.61)

Note: Ham-A = Hamilton Rating Scale for Anxiety. Ham-D = Hamilton Rating Scale for Depression. PSWQ = Penn State Worry Questionnaire. IUS = Intolerance of Uncertainty Scale.

### Procedures

As illustrated in S1 Fig., each trial began with an anticipatory cue presented for two seconds. An ‘X’ signified that an aversive picture would be presented, ‘O’ preceded neutral pictures, and ‘?’ indicated that either could be presented (half were followed by aversive and half by neutral pictures). Participants were instructed about all cue-picture pairings prior to scanning. The cue was followed by an intertrial interval (ITI) of 2–8 s, then an aversive or neutral picture from the International Affective Picture System [50], another ITI of 5–9 s, then a 5 s rating period (S1 Fig.), and a final ITI of 1–5 s. Cues were white on a black background, and ITIs included a fixation cross. Trial order was pseudo-randomized, with the stipulation that no trial type (aversive, neutral, or uncertain) was presented more than twice in a row. Across four functional scan runs, there were 38 aversive pictures presented after ‘X’ cues, 38 neutral pictures presented after ‘O’ cues, and 19 of each after ‘?’ cues, with all pictures presented only once. Aversive pictures were the most unpleasant and arousing in the picture set (e.g., mutilated bodies, attack scenes), based on published norms [50]. Pictures with neutral valence and low arousal ratings comprised the neutral pictures (e.g., household items).

**DNA**. DNA was extracted from buccal cells obtained from 10-ml rinse of commercial mouthwash, using the Gentra Puregene DNA purification system (Qiagen cat no. 158867). Genotypes for 5-HTTLPR (S and L alleles) and for rs25531 (L_A_ and L_G_ alleles) were determined using an established protocol [51]. Participants with L_G_ alleles were grouped with those expressing S alleles given evidence of nearly equivalent 5-HTT expression for these variants [6].

**MRI Data Acquisition, Processing, and Analysis**. Magnetic resonance images were acquired on a 3.0 Tesla scanner (GE Signa; Waukesha, WI) with a high-speed, whole-body gradient and whole-head transmit-receive coil (4 channel). Whole-brain high-resolution T1-weighted anatomical scans (axial acquisition, 3D, inversion-recovery spoiled gradient echo: FOV 240 mm, matrix 256x192, in-plane resolution 0.9 mm, 124 1.2-mm axial slices, 10° flip angle) were acquired contiguous with functional scans for aid in registration and normalization of functional data. Functional data (2D gradient echo [GRE] planar) consisted of 30 interleaved slices (slice thickness/gap: 4mm/1mm) acquired sagittally (TR = 2000 ms, TE = 30 ms, 90° flip angle) resulting in 64x64 matrix data with 240 mm FOV, and 3.75mm in-plane resolution. A Silent Vision system (Avotec, Inc., Jensen Beach, FL) displayed visual stimuli via stereoscopic goggles mounted on the head coil.

Processing of fMRI data was done using AFNI (http://afni.nimh.nih.gov/afni/) version 2.56a starting wtih slice-time correction, motion correction (6-parameter rigid body), fieldmap correction (GRE using sagittal acquisition; TE = 7 and 10 ms; TR = 700 ms) [52] then high pass temporal filtering (128s). Images from the four functional scan runs were aligned to the run closest in time to the high-resolution anatomical scan. To minimize the influence of magnetization equilibrium, the first three volumes of each scan were discarded. Event-related data were analyzed as percent change (vs. baseline) calculated for each subject. Events were modeled at the first-level using least-squares regression to fit the hemodynamic response function to a gamma variate for anticipation and response periods, separately [23], as well as the rating period (included in model but not analyzed here) all specified by aversive and neutral valence. Residuals from motion correction (6 directions) were included in the general linear model as regressors of no interest, and the baseline was modeled using a 5th order polynomial function. Each subject’s T1 scan was AC/PC aligned and normalized to the MNI 152 brain using a 12-parameter affine transformation (@auto_tlrc). Functional images were coregistered to the T1 in native space and in the same step normalized to MNI using the T1 derived algorithms. Alignment between functional and T1 as well as between warped data and MNI were visually inspected to confirm accurate registration.

Percent signal change values averaged over voxels in *a priori* regions of interest (ROI) for bilateral amygdala and anterior insula were extracted for each condition of interest (AFNI 3dmaskave; non-zero voxels). Both ROIs were defined using the Talairach Daemon atlas [53], with the additional specification for the anterior insula to only include voxels between y = 24 and y = -2. Study hypotheses were tested with diagnostic Group (patient, control) x Genotype (S/L_G_ carriers, L_A_ homozygotes) x Period (anticipation, picture) x Valence (aversive, neutral) ANOVAs for the amygdala and for the anterior insula ROI using SPSS (version 21). Analyses including the uncertain trials did not alter the hypothesized Group x Genotype findings and are not reported. No violations to assumptions were detected with Mauchly’s test for assumption of sphericity (ANOVA) whereas degrees of freedom adjustments were made with significant findings from Levene’s test for violations of homoscedasticity assumptions (*t*-tests). Pearson’s correlations tested for associations between activity in each ROI and number of L_A_ alleles as follow up tests. Amygdala and insula associations with symptom measures (Ham-A, Ham-D, PSWQ, and IUS) were assessed in simultaneous regressions for relationships in patient data. For primary results, partial eta square for ANOVA and Hedges’ unbiased measure of *g* [54] for *t*-tests are reported as effect size measures $\eta_{p}^{2}$ and *g*, respectively.

The above ROI-based analyses were supplemented with secondary voxelwise tests given evidence of functional and cellular heterogeneities across the amygdala [55,56] and insula [25,57] that may be more or less sensitive to our effects of interest. The voxelwise ANOVA testing the hypothesized Group x Gene interaction employed a tool for group analysis with unbalanced sample sizes (AFNI GroupAna). Voxelwise regressions were conducted for number of L_A_ alleles and for symptom measures. All voxelwise analyses were conducted beginning with uncorrected *p*<.005 and thresholded to *p*<0.05 corrected using small-volume (separate for bilateral amygdala and also for bilateral anterior insula) correction for multiple comparisons which resulted in a minimum cluster size of 240 mm^3^ for the amygdala and 504 mm^3^ for the anterior insula (AFNI AlphaSim). Figures display *p*<.005 uncorrected images thresholded for cluster sizes corresponding to a corrected *p*<.05.

## Results

### Descriptives

Fisher’s exact test indicated no genotype differences by group, *p* = 0.634 (Table 1). Across groups, L_A_ homozygotes were slightly younger (*M* = 21.91 vs. 27.67), *t*(76.73) = -3.769, *p* = 0.001, and less educated (*M* = 14.65 vs. 16.27 years) than S/L_G_ carriers, *t*(87) = -3.76, *p* = 0.001. Across genotype, patients did not differ from healthy comparison subjects on age, *t*(85.66) = 1.52, *p* = 0.132, or education, *t*(87) = -1.90, *p* = 0.061. Also, there was no interaction between genotype and patient status on age, *F*(1,85) = 0.02, *p* = 0.880 or education, *F*(1,85) = 0.53, *p* = 0.466. As expected, patients and healthy subjects differed on the Ham-A, Ham-D, PSWQ, and IUS (*p*s<0.001), but there were no genotype differences on those instruments within each group (*p*s>0.120). Participants in each group had allelic frequencies in Hardy-Weinberg equilibrium (*p*s>0.600). Within the patient group, there were no differences by genotype in the number of comorbid disorders (L_A_: *M* = 0.93; S/L_G_: *M* = 0.92), *t*(48) = 0.05, *p* = 0.962. The ethnic composition of the sample was 5.6% African American, 12.4% Asian, and 79.8% Caucasian (1.1% ethnicity not reported). For primary results, each ethnic group was systematically evaluated as a potential outlier (see below) according to genotype and neural response. Neither group nor genotype varied systematically by ethnic identification (*p*s>0.61). The patient group had a larger proportion of women than the control group, *Χ* ^2^(1) = 4.81, *p* = 0.028, but there was no gender disparity for genotype, *Χ* ^2^(1) = 0.81, *p* = 0.368.

### Amygdala Effects

**Fig 1:**
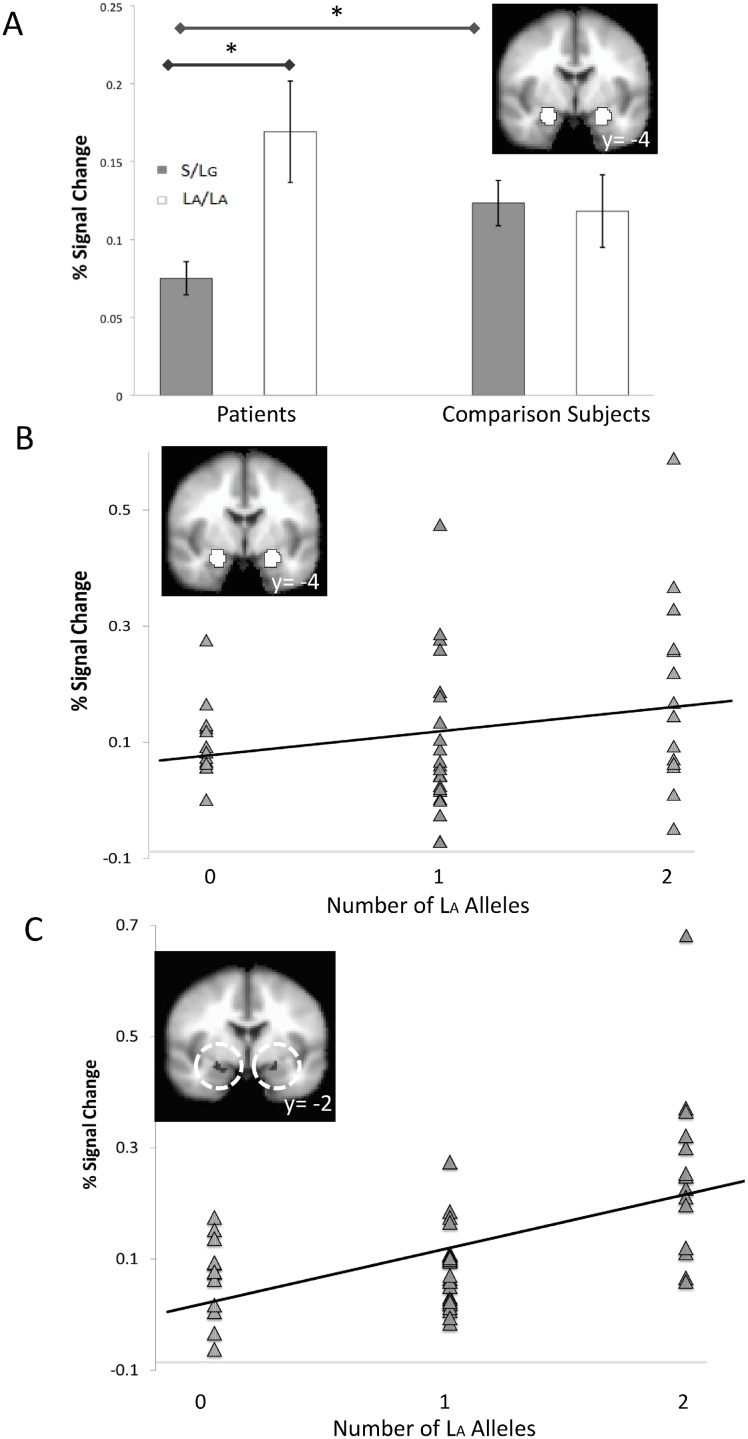
5-HTTLPR genotype effects on amygdala activation differentiate patients and healthy comparison subjects. A) Patients with at least one S or L_G_ allele (*n* = 36) showed less amygdala activation than patients with two L_A_ alleles (*n* = 14) and than healthy comparison subjects with at least one S or L_G_ allele (*n* = 30). This Group x Genotype effect of 5-HTTLPR was observed in amygdala *a priori* regions of interest (inset) across aversive and neutral valences in anticipation of and response to affective pictures. B) A confirmatory finding in patients (n = 50) indicated a positive association between the number of L_A_ alleles of the serotonin transporter gene and bilateral activation in the amygdala *a priori* region of interest (inset). C) A voxelwise regression for patients indicated a positive association between L_A_ allele number and bilateral amygdala activation (inset) at *p*<0.05, corrected. Y-axes display mean percent signal change averaged over displayed ROIs/clusters. Asterisks on the bar graph indicate significant differences at *p*<0.05. Y-values on coronal images indicate Tailarach and Tournoux coordinates for the AFNI MNI-152 brain used for normalizing. Error bars are mean standard errors.

For the amygdala, a Valence main effect across anticipation and picture periods replicated previous findings of greater amygdala activation to aversive than neutral trials, *F*(1,85) = 10.09, *p* = 0.002 [20,21,22], serving as a manipulation check. A Group x Genotype interaction for the amygdala, *F*(1,85) = 6.17, *p* = 0.015, $\eta_{p}^{2}$ = 0.07, revealed that S/L_G_-carrier patients had less amygdala activation than L_A_/L_A_ patients, *t*(48) = 3.58, *p* = 0.001, *g* = 0.81, and than S/L_G_-carrier comparison subjects, *t*(64) = -2.74, *p* = 0.008, *g* = 0.67 (Fig. 1A). Genotype differences previously reported in healthy samples [10] were not replicated within the comparison subjects, *t*(37) = -0.18, *p* = 0.859, *g* = 0.07, perhaps due to a failure to detect the small effect size estimated for this contrast [10]. In addition, patients showed an association between number of L_A_ alleles and amygdala activity, *r* = 0.39, *p* = 0.005, whereas the healthy comparison subjects did not, *r* = -0.04, *p* = 0.808, correlations that differed significantly from one another (Fisher r-to-z) *z* = -2.06, *p* = 0.047 (Fig. 1B). The findings were nearly identical for partial correlations controlling for age and years of education (patients *r* = 0.37, *p* = 0.010; controls *r* = -0.04, *p* = 0.815; *z* = -1.93, *p* = 0.027). These data were confirmed by voxelwise regressions at *p*<0.05 (corrected) for the right amygdala and marginally for the left (Fig. 1C; Table 2).

Across groups, amygdala effects for Genotype, *F*(1,85) = 4.93, *p*<0.001, Period, *F*(1,85) = 3.36, *p*<0.001, Valence, *F*(1,85) = 10.09, *p* = 0.002, and Period x Genotype, *F*(1,85) = 4.60, *p* = 0.035, were qualified by a three-way Genotype x Period x Valence interaction, *F*(1,85) = 12.49, *p* = 0.001. Posthoc analyses indicated a significant Genotype x Valence interaction for the picture period, *F*(1,85) = 6.03, *p* = 0.015, but not for the anticipation period, *F*(1,85) = 1.41, *p* = 0.110. L_A_/L_A_ homozygotes showed greater amygdala responses than S/L_G_ carriers to aversive pictures, *t*(87) = 3.94, *p*<0.001, but not to neutral pictures, *t*(87) = 0.89, *p* = 0.376. There were no other effects for ROI-based analyses on the amygdala, and Group x Genotype findings for the amygdala did not attain the *p*<0.05 (corrected) threshold of significance for voxelwise ANOVAs.

### Anterior Insula Effects

**Fig 2:**
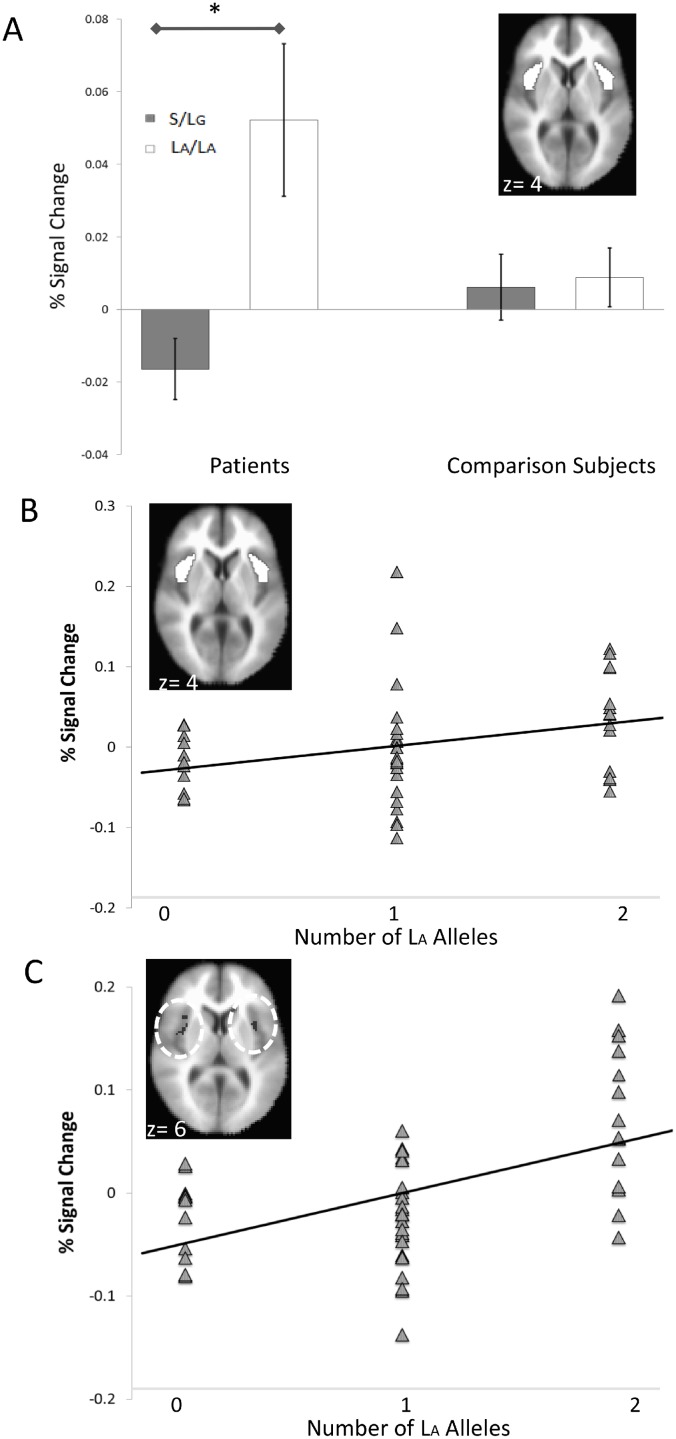
5-HTTLPR genotype effects on anterior insula activation differentiate patients and healthy comparison subjects. A) Patients with at least one S or L_G_ allele (*n* = 36) showed less anterior insula activation than patients with two L_A_ alleles (*n* = 14). This Group x Genotype effect of 5-HTTLPR was observed in anterior insula *a priori* regions of interest (inset) across aversive and neutral valences in anticipation of and response to affective pictures. B) A confirmatory finding in patients (*n* = 50) indicated a positive association between the number of L_A_ alleles of the serotonin transporter gene and bilateral activation in anterior insula *a priori* region of interest (inset). C) A voxelwise regression for patients indicated a positive association between L_A_ allele number and bilateral anterior insula activation (inset) at *p*<0.05, corrected. Y-axes display mean percent signal change averaged over displayed ROIs/clusters. Asterisks on the bar graph indicate significant differences at *p*<0.05. Y-values on axial images indicate Tailarach and Tournoux coordinates for the AFNI MNI-152 brain used for normalizing. Error bars are mean standard errors.

Similar effects were observed for the anterior insula. The manipulation check testing the Valence main effect across anticipation and picture periods, *F*(1,85) = 13.93, *p*<0.001, replicated previous findings for the anterior insula [20,21,22]. The Group x Genotype interaction, *F*(1,85) = 6.18, *p* = 0.015, $\eta_{p}^{2}$ = 0.07, indicated that S/L_G_-carrier patients had less insula activation than L_A_/L_A_ patients, *t*(17.29) = 3.03, *p* = 0.007, *g* = 1.14 (Fig. 2A), whereas comparison subjects did not differ by genotype, *t*(28.38) = 0.23, *p* = 0.823, *g* = 0.06. Patients showed an association between number of L_A_ alleles and insula activity, *r* = 0.30, *p* = 0.034, whereas comparison subjects did not, *r* = -0.06, *p* = 0.733 (Fig. 2B), correlations that also yielded a one-tailed group difference, *z* = -1.67, *p* = 0.048. The findings were essentially identical for partial correlations controlling for age and years of education (patients *r* = 0.30, *p* = 0.042; controls *r* = -0.08, *p* = 0.644; *z* = -1.73, *p* = 0.039). Similar to the amygdala, correlations with the insula ROIs were confirmed by voxelwise regressions at *p*<0.05 (corrected), which revealed bilateral anterior insula effects (Fig. 2C; Table 2).

Across groups, anterior insula effects for Genotype, *F*(1,85) = 7.25, *p* = 0.009, Period, *F*(1,85) = 18.81, *p*<0.001, Valence, *F*(1,85) = 13.93, *p*<0.001, and Period x Genotype, *F*(1,85) = 4.86, *p* = 0.030, were qualified by a three-way Genotype x Period x Valence interaction, *F*(1,85) = 8.49, *p* = 0.005. Similar to the amygdala, posthoc analyses indicated a significant Genotype x Valence interaction for the picture period, *F*(1,85) = 4.99, *p* = 0.028, but not for the anticipation period, *F*(1,85) = 0.32, *p* = 0.572. L_A_/L_A_ homozygotes showed greater insula responses than S/L_G_ carriers to aversive pictures, *t*(87) = 4.16, *p*<0.001, but not to neutral pictures, *t*(87) = 1.35, *p* = 0.180. There were no other effects for the anterior insula, and Group x Genotype findings did not attain the *p*<0.05 (corrected) threshold of significance for voxelwise ANOVAs.

### Additional Regions

To test the specificity of similar findings observed for the amygdala and anterior insula, we conducted exploratory ROI analyses for other areas implicated in prior work on anticipating and responding to aversive pictures [20,21,22,58]. The Group x Genotype effect was not observed for the pregenual anterior cingulate (pACC), anterior mid-cingulate (aMCC), or hippocampus for ROI-based analyses (all *p*s>0.19) or for whole-brain voxelwise analyses at *p*<0.05 (corrected) or *p*<0.005 (uncorrected).

### Symptom Correlates

**Fig 3:**
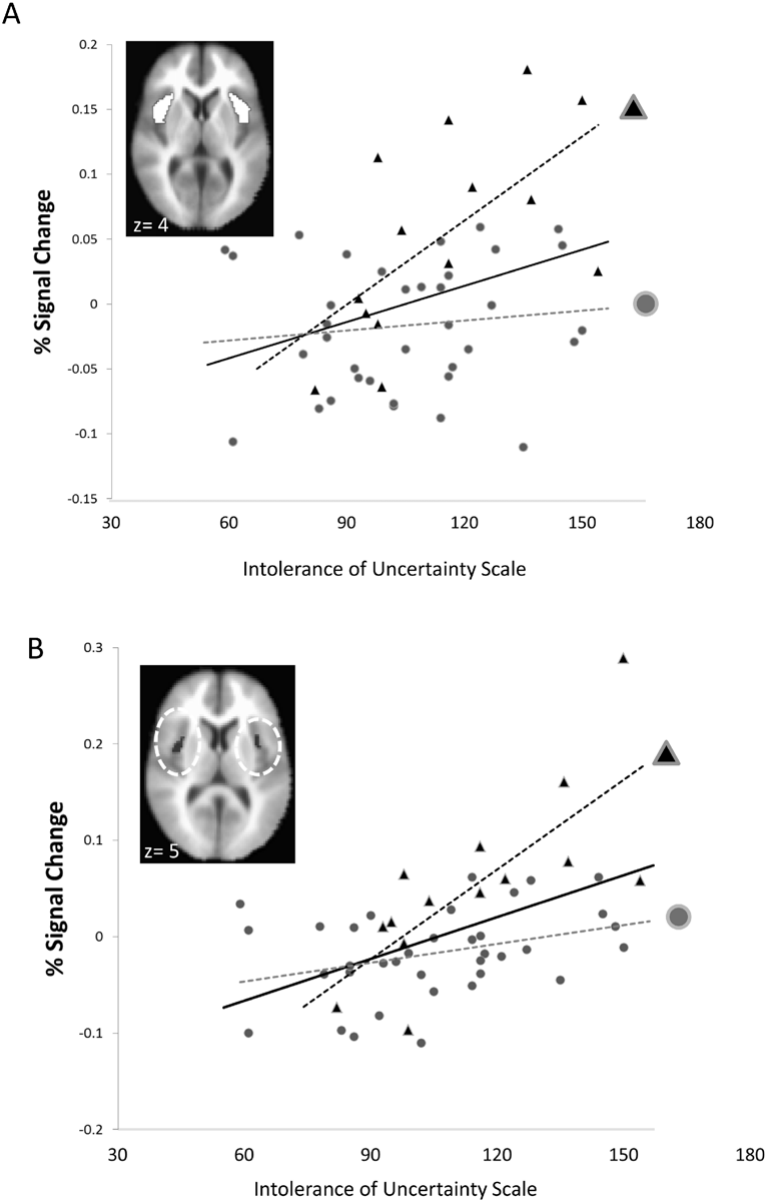
5-HTTLPR genotype effects on association between anterior insula activation and intolerance of uncertainty in patients. Patients with two L_A_ alleles (*n* = 14) and higher scores on the Intolerance of Uncertainty Scale (IUS) had more anterior insula activation across aversive and neutral valences in anticipation of and response to affective pictures in the *a priori* defined anterior insula region of interest (inset). B) A voxelwise regression for patients with two L_A_ alleles indicated a positive association between the IUS and bilateral anterior insula activation (inset) at *p*<0.05, corrected. No associations with the IUS were observed for patients with at least one S or L_G_ allele (*n* = 36). The association remained significant even after removing the individual with the highest insula activation, *r* = 0.44, *p* = 0.001. Y-axes display mean percent signal change averaged over displayed ROIs/clusters. Solid regression lines indicate all patients, triangular markers and black dashed lines indicate patients with two L_A_ alleles, and circular markers and gray dashed lines indicate patients who are S/L_G_ carriers. Z-values on axial images indicate Tailarach and Tournoux coordinates for the AFNI MNI-152 brain used for normalizing.

Within the patient group, simultaneous regression analyses with the four symptom measures as predictors revealed that L_A_/L_A_ patients showed an association between activity in the *a priori* anterior insula ROIs and the IUS, s*ß* = 0.73, *p* = 0.029 (zero-order Pearson’s correlation, *r* = 0.62, *p* = 0.018) (Fig. 3A). The anterior insula association with the IUS was also present for patients when collapsing across 5-HTTLPR status, s*ß* = 0.37, *p* = 0.024, but not for patients who were S/L_G_ carriers, s*ß* = 0.11, *p* = 0.584. The insula/IUS correlation was also significant as a partial correlation controlling for the other scales (PSWQ, HamA, HamD) in the full patient group (*r* = 0.33, *p* = 0.024) and in the L_A_/L_A_ group (*r* = 0.65, *p* = 0.029), but not in the S/L_G_ carriers (*r* = 0.10, *p* = 0.584). A voxelwise regression at *p*<0.05 (corrected) revealed the same association with the IUS in bilateral insula for the L_A_/L_A_ subgroup of patients, as well as for the patients overall (Fig. 3B). No significant correlations with amygdyala or additional significant correlations between anterior insula activity and self-report were observed for either genotype subgroup using ROI-based or voxelwise analyses at the above corrected thresholds.

### Ethnicity

For each group (patient/non; high/low gene expression), there were no outliers (2 SDs difference) according to ethnic group (African American/Non; Asian/Non; No Ethnicity Reported/Reported) with the exception of one African American control participant with the low SERT expressing genotype who had excessively high amygdala responses. Removing this data point did not alter the within-group or between-group effects reported above.

## Discussion

Evidence suggests that genes do not encode for specific psychiatric illnesses or even for specific psychiatric symptoms. However, evidence is mounting that common genetic polymorphisms play important roles in the development of affective disorders, stress reactivity, and responsiveness to treatment [5,10,15,16,59]. In the present study, the impact of serotonin transporter genotype on affective neural responses distinguished patients with clinical anxiety (GAD; some of whom were also diagnosed with SAD and/or MDD) from healthy non-psychiatric participants. In both the amygdala and anterior insula, patients with two L_A_ alleles exhibited greater activation across anticipatory cue and affective picture presentations than patients who are S/L_G_ carriers. Moreover, the patients showed an association between greater amygdala and insula responses and the number of L_A_ alleles. Finally, the pattern of neural responding in patients correlated with self-reported intolerance of uncertainty, a central feature across multiple affective disorders [45,60].

The activation patterns found here are consistent with a previous neuroimaging study of 5-HTTLPR genotype and amygdala responding in another mixed patient group including GAD, SAD, separation anxiety disorder, and MDD [11] but divergent from a sample reported as MDD but lacking details on psychiatric comorbidity [12]. In the first study, patients with the L_A_/L_A_ genotype had greater amygdala activity than S/L_G_ carriers for both fear and happy face presentations in an adolescent sample [11]. In the second study, the number of low expressing alleles in MDD patients was associated positively with amygdala response to affective pictures [12]. Our results extend these patient data to an adult sample anticipating and then viewing affective pictures, specifying additional clinical characteristics, and also replicating amygdala patterns in the anterior insula. The present study is also one of a growing number of neuoroimaging genetics studies to use the critically important triallelic classification in patient samples [11,12,13,17]. Potential mechanisms for 5-HTTLPR genotype effects remain an area of active investigation [15,61,62], but these data add to the growing corpus of literature that suggest greater complexity in how the 5-HTTLPR genotype influences affective neural circuitry, especially in psychiatric disorders. The results suggest that the low expressing 5-HTTLPR variant does not, in all cases, lead to greater responsivity in affective neural systems. A wide range of deleterious as well as protective mood and environmental factors could alter functional trajectories in 5-HTTLPR variant subtypes [5] which should be examined further in relation to our findings.

The functional significance of genotype effects warrants careful consideration of specificity and context. Anatomic specificity for the amygdala and insula in the current report was ascertained by evaluating reactivity in other key brain areas implicated in work on emotion and psychopathology. 5-HTTLPR genotype effects were not observed for the ACC, aMCC, or hippocampus. Conversely, there was an absence of predicted specificity with respect to findings for particular components of the fMRI task employed. The pattern of reduced amygdala and insula responses for patients with at least one S or L_G_ allele was observed across the anticipation and picture periods and was equivalent for aversive and neutral trials. Indeed, we did not extend our previous findings of increased anticipatory amygdala activity in GAD patients without other Axis I comorbidities [23] to the present group of mostly comorbid GAD patients. Because the previous study did not include DNA collection, we could not ascertain whether genotype differences contributed to the discrepant findings for anticipatory activity. Also of relevance is the recent discussion of statistical power in neuroscience research [63] noted above as a potential explanation for not replicating the 5-HTTLPR genotype effect in amygdala activity reported in healthy populations [10]. Given a wealth of published work supporting greater affective responsivity in healthy individuals with short alleles, our lack of replication for this effect in our control sample is probably best explained by low statistical power. For future studies, if recruitment begins with assays of genotype before the imaging acquisition, participants can be pre-selected to fill these smaller cells and increase power in detecting neurobiological effects. The pattern in patients, on the other hand, was of a sufficient magnitude to yield statistical significance. Given the strength of the patient findings, it is evident that 5-HTTLPR genotype effects are particularly pronounced in patients but have a different impact on neural response to affective stimuli as compared with weaker effects observed in healthy samples.

To put these findings in context, it is helpful to consider the dynamic interplay of processes that may be involved in the development of affective disorders along with limitations of the present study. Risk for development of clinically significant anxiety and depression involves many genes, complex interactions with life stress, and depends on developmentally dynamic biological processes. Stressful life events may influence physiological reactivity as determined by complex interactions between serotonin transporter genotype and age, type of stressful experience, and number of stressful experiences [61]. Genetic contributions to neural responsivity may similarly set the stage for dysregulated affective responding by contributions to an intermediate phenotype the ultimate consequence of which is determined by experiential factors [64,65]. Amygdala responding to fearful faces is also greater for children thought to be at risk for developing major depression given their family histories, consistent with the intermediate phenotype hypothesis [66]. Similarly, healthy participants with elevated anxiety levels (‘anxiety prone’) show greater levels of amygdala and insula activation to emotional faces [67], perhaps as a function of genetic risk but without stressful life event contributions to induce full-blown disorders. Our results do not yield a genetic mechanism for development of affective disorders. Instead, they suggest a specific impact on neurobiology among patients that subdivides this group into hyper or hypo amygdala and insula responsivity and that may resolve apparent differences across studies, especially in GAD [23,37]. There may be additional experimental paradigms that uncover similar or different results from those found here such as those that rely more on reward processing as being more directly relevant to depression. Having additional participants with single disorders (GAD or MDD or SAD) would also help to test potential differences by diagnostic group. Finally, we did not take histories of stressful life events that would have been informative on the course of illness development and contributions of life events to observed neural group differences which should be a focus of future studies.

### Conclusion

Using a triallelic polymorphism (5-HTTLPR and rs25531) to investigate genetic variation in serotonin transporter expression among patients free of medications that might influence serotonin activity in the brain, we establish evidence for the influence of serotonin transporter gene variants on amygdala and anterior insula activity in pathology spanning GAD, SAD, and MDD. Importantly, serotonin transporter expression influences on activity in affective neurocircuitry may diverge in pathological and healthy individuals, as shown here in adults and previously in a mixed sample of adolescent patients [11]. Neural profiles that include genetic subtypes could serve as endophenotypic markers of disorders, symptom clusters, or psychopathology dimensions, and inform pioneering efforts in the development of individualized treatment regimens and personalized medicine.

## Supporting Information

S1 Fig The experimental paradigm. The experimental paradigm included three types of warning cues (X, O, ?). The ‘X’ cue indicated that an aversive picture would be presented; the ‘O’ cue indicated that a neutral picture would be presented; the ‘?’ cue indicated an equal probability of either an aversive or neutral picture. Warning cues were presented for two seconds. Pictures from the International Affective Picture System (IAPS) were presented for one second. Ratings of pictures or mood (50% of each, counterbalanced by picture valence) were presented for 5 seconds following each picture. ISIs between cue and picture ranged from 4–10 seconds, between picture and rating scale varied between 5–9 seconds, and between rating scale and subsequent warning cue ranged between 1–5 seconds. Aversive and neutral pictures were equated for luminance, and men and women viewed slightly different aversive picture sets based on normative ratings (36). Pictures shown below are not from IAPS and are for illustration purposes only. In addition to the 114 cue-picture trials described in the text, there were 14 catch trials for which no picture was presented and 12 catch trials for which a picture was presented with no warning cue (counterbalanced by valence). The fMRI data from these catch trials were not analyzed for this report. On the catch trials without a picture, a rating scale for anticipatory anxiety was presented for 5 s. Using a response box during the fMRI experiment, participants provided mood or picture ratings after the presentation of all pictures on an 11-point rating scale (-5 for “unpleasant,” 0 for “neutral,” 5 for “pleasant”) and anticipatory anxiety ratings after the cues on catch trials without a picture (0 for “Not at all,” 4 for “Moderately,” 8 for “Extremely”). Approximately one week before the fMRI experimental session, all subjects were positioned in a mock scanner, including head coil, goggles, and response box. After being instructed about all cue-picture pairings, subjects viewed an abbreviated version of the experimental paradigm, using pictures not shown during the experimental session.
(TIF)

## References

[1] Regier DA, Kuhl EA, Kupfer DJ (2013) The DSM-5: Classification and criteria changes. World Psychiatry 12: 92–98. 10.1002/wps.20050 23737408 PMC3683251

[2] Nemeroff CB, Weinberger D, Rutter M, MacMillan HL, Bryant RA, et al (2013) DSM-5: a collection of psychiatrist views on the changes, controversies, and future directions. BMC Med 11: 202 10.1186/1741-7015-11-202 24229007 PMC3846446

[3] Insel T, Cuthbert B, Garvey M, Heinssen R, Pine DS, et al (2010) Research domain criteria (RDoC): toward a new classification framework for research on mental disorders. Am J Psychiatry 167: 748–751. 10.1176/appi.ajp.2010.09091379 20595427

[4] Jacobs BL, Azmitia EC (1992) Structure and function of the brain serotonin system. Physiol Rev 72: 165–229. 1731370 10.1152/physrev.1992.72.1.165

[5] Caspi A, Hariri AR, Holmes A, Uher R, Moffitt TE (2010) Genetic sensitivity to the environment: the case of the serotonin transporter gene and its implications for studying complex diseases and traits. Am J Psychiatry 167: 509–527. 10.1176/appi.ajp.2010.09101452 20231323 PMC2943341

[6] Hu XZ, Lipsky RH, Zhu G, Akhtar LA, Taubman J, et al (2006) Serotonin transporter promoter gain-of-function genotypes are linked to obsessive-compulsive disorder. Am J Hum Genet 78: 815–826. 16642437 10.1086/503850 PMC1474042

[7] Li Q (2006) Cellular and molecular alterations in mice with deficient and reduced serotonin transporters. Mol Neurobiol 34: 51–66. 17003521 10.1385/mn:34:1:51

[8] Lim JE, Papp A, Pinsonneault J, Sadee W, Saffen D (2006) Allelic expression of serotonin transporter (SERT) mRNA in human pons: lack of correlation with the polymorphism SERTLPR. Mol Psychiatry 11: 649–662. 16432527 10.1038/sj.mp.4001797

[9] Parsey RV, Hastings RS, Oquendo MA, Hu X, Goldman D, et al (2006) Effect of a triallelic functional polymorphism of the serotonin-transporter-linked promoter region on expression of serotonin transporter in the human brain. Am J Psychiatry 163: 48–51. 16390888 10.1176/appi.ajp.163.1.48

[10] Murphy SE, Norbury R, Godlewska BR, Cowen PJ, Mannie ZM, et al (2013) The effect of the serotonin transporter polymorphism (5-HTTLPR) on amygdala function: a meta-analysis. Mol Psychiatry 18: 512–520. 10.1038/mp.2012.19 22488255

[11] Lau JY, Goldman D, Buzas B, Fromm SJ, Guyer AE, et al (2009) Amygdala function and 5-HTT gene variants in adolescent anxiety and major depressive disorder. Biol Psychiatry 65: 349–355. 10.1016/j.biopsych.2008.08.037 18950748 PMC2791528

[12] Friedel E, Schlagenhauf F, Sterzer P, Park SQ, Bermpohl F, et al (2009) 5-HTT genotype effect on prefrontal-amygdala coupling differs between major depression and controls. Psychopharmacology (Berl) 205: 261–271. 10.1007/s00213-009-1536-1 19387615

[13] Dannlowski U, Ohrmann P, Bauer J, Deckert J, Hohoff C, et al (2008) 5-HTTLPR biases amygdala activity in response to masked facial expressions in major depression. Neuropsychopharmacology 33: 418–424. 17406646 10.1038/sj.npp.1301411

[14] Hariri AR, Mattay VS, Tessitore A, Kolachana B, Fera F, et al (2002) Serotonin transporter genetic variation and the response of the human amygdala. Science 297: 400–403. 12130784 10.1126/science.1071829

[15] Canli T, Omura K, Haas BW, Fallgatter A, Constable RT, et al (2005) Beyond affect: a role for genetic variation of the serotonin transporter in neural activation during a cognitive attention task. Proc Natl Acad Sci U S A 102: 12224–12229. 16093315 10.1073/pnas.0503880102 PMC1189322

[16] Pergamin-Hight L, Bakermans-Kranenburg MJ, van Ijzendoorn MH, Bar-Haim Y (2012) Variations in the promoter region of the serotonin transporter gene and biased attention for emotional information: a meta-analysis. Biol Psychiatry 71: 373–379. 10.1016/j.biopsych.2011.10.030 22138391

[17] Dannlowski U, Ohrmann P, Bauer J, Kugel H, Baune BT, et al (2007) Serotonergic genes modulate amygdala activity in major depression. Genes Brain Behav 6: 672–676. 17284168 10.1111/j.1601-183X.2006.00297.x

[18] Domschke K, Braun M, Ohrmann P, Suslow T, Kugel H, et al (2006) Association of the functional-1019C/G 5-HT1A polymorphism with prefrontal cortex and amygdala activation measured with 3 T fMRI in panic disorder. Int J Neuropsychopharmacol 9: 349–355. 16316476 10.1017/S1461145705005869

[19] Furmark T, Tillfors M, Garpenstrand H, Marteinsdottir I, Langstrom B, et al (2004) Serotonin transporter polymorphism related to amygdala excitability and symptom severity in patients with social phobia. Neurosci Lett 362: 189–192. 15158011 10.1016/j.neulet.2004.02.070

[20] Nitschke JB, Sarinopoulos I, Mackiewicz KL, Schaefer HS, Davidson RJ (2006) Functional neuroanatomy of aversion and its anticipation. Neuroimage 29: 106–116. 16181793 10.1016/j.neuroimage.2005.06.068

[21] Grupe DW, Oathes DJ, Nitschke JB (2013) Dissecting the anticipation of aversion reveals dissociable neural networks. Cereb Cortex 23: 1874–1883. 10.1093/cercor/bhs175 22763169 PMC3698367

[22] Sarinopoulos I, Grupe DW, Mackiewicz KL, Herrington JD, Lor M, et al (2010) Uncertainty during anticipation modulates neural responses to aversion in human insula and amygdala. Cereb Cortex 20: 929–940. 10.1093/cercor/bhp155 19679543 PMC2837092

[23] Nitschke JB, Sarinopoulos I, Oathes DJ, Johnstone T, Whalen PJ, et al (2009) Anticipatory activation in the amygdala and anterior cingulate in generalized anxiety disorder and prediction of treatment response. Am J Psychiatry 166: 302–310. 10.1176/appi.ajp.2008.07101682 19122007 PMC2804441

[24] Etkin A, Wager TD (2007) Functional neuroimaging of anxiety: a meta-analysis of emotional processing in PTSD, social anxiety disorder, and specific phobia. Am J Psychiatry 164: 1476–1488. 17898336 10.1176/appi.ajp.2007.07030504 PMC3318959

[25] Kurth F, Zilles K, Fox PT, Laird AR, Eickhoff SB (2010) A link between the systems: functional differentiation and integration within the human insula revealed by meta-analysis. Brain Struct Funct 214: 519–534. 10.1007/s00429-010-0255-z 20512376 PMC4801482

[26] Nitschke JB, Heller W., Imig J.C., McDonald R.P., Miller G.A. (2001) Distinguishing dimensions of anxiety and depression. Cognitive Therapy and Research 25: 1–22.

[27] LeDoux JE (2000) Emotion circuits in the brain. Annu Rev Neurosci 23: 155–184. 10845062 10.1146/annurev.neuro.23.1.155

[28] Debiec J, Sullivan RM (2014) Intergenerational transmission of emotional trauma through amygdala-dependent mother-to-infant transfer of specific fear. Proc Natl Acad Sci U S A 111: 12222–12227. 10.1073/pnas.1316740111 25071168 PMC4142995

[29] Johansen JP, Hamanaka H, Monfils MH, Behnia R, Deisseroth K, et al (2010) Optical activation of lateral amygdala pyramidal cells instructs associative fear learning. Proc Natl Acad Sci U S A 107: 12692–12697. 10.1073/pnas.1002418107 20615999 PMC2906568

[30] Admon R, Milad MR, Hendler T (2013) A causal model of post-traumatic stress disorder: disentangling predisposed from acquired neural abnormalities. Trends Cogn Sci 17: 337–347. 10.1016/j.tics.2013.05.005 23768722

[31] Paulus MP, Stein MB (2006) An insular view of anxiety. Biol Psychiatry 60: 383–387. 16780813 10.1016/j.biopsych.2006.03.042

[32] Simmons AN, Stein MB, Strigo IA, Arce E, Hitchcock C, et al (2011) Anxiety positive subjects show altered processing in the anterior insula during anticipation of negative stimuli. Hum Brain Mapp 32: 1836–1846. 10.1002/hbm.21154 21181800 PMC3215249

[33] Seeley WW, Menon V, Schatzberg AF, Keller J, Glover GH, et al (2007) Dissociable intrinsic connectivity networks for salience processing and executive control. J Neurosci 27: 2349–2356. 17329432 10.1523/JNEUROSCI.5587-06.2007 PMC2680293

[34] Acevedo BP, Aron EN, Aron A, Sangster MD, Collins N, et al (2014) The highly sensitive brain: an fMRI study of sensory processing sensitivity and response to others’ emotions. Brain Behav 4: 580–594. 10.1002/brb3.242 25161824 PMC4086365

[35] Craig AD (2011) Significance of the insula for the evolution of human awareness of feelings from the body. Ann N Y Acad Sci 1225: 72–82. 10.1111/j.1749-6632.2011.05990.x 21534994

[36] McClure EB, Monk CS, Nelson EE, Parrish JM, Adler A, et al (2007) Abnormal attention modulation of fear circuit function in pediatric generalized anxiety disorder. Arch Gen Psychiatry 64: 97–106. 17199059 10.1001/archpsyc.64.1.97

[37] Etkin A, Prater KE, Hoeft F, Menon V, Schatzberg AF (2010) Failure of anterior cingulate activation and connectivity with the amygdala during implicit regulation of emotional processing in generalized anxiety disorder. Am J Psychiatry 167: 545–554. 10.1176/appi.ajp.2009.09070931 20123913 PMC4367202

[38] Hamilton JP, Etkin A, Furman DJ, Lemus MG, Johnson RF, et al (2012) Functional neuroimaging of major depressive disorder: a meta-analysis and new integration of base line activation and neural response data. Am J Psychiatry 169: 693–703. 10.1176/appi.ajp.2012.11071105 22535198 PMC11889638

[39] Blair K, Shaywitz J, Smith BW, Rhodes R, Geraci M, et al (2008) Response to emotional expressions in generalized social phobia and generalized anxiety disorder: evidence for separate disorders. Am J Psychiatry 165: 1193–1202. 10.1176/appi.ajp.2008.07071060 18483136 PMC2855133

[40] Goldin PR, Manber T, Hakimi S, Canli T, Gross JJ (2009) Neural bases of social anxiety disorder: emotional reactivity and cognitive regulation during social and physical threat. Arch Gen Psychiatry 66: 170–180. 10.1001/archgenpsychiatry.2008.525 19188539 PMC4142809

[41] Keedwell PA, Drapier D, Surguladze S, Giampietro V, Brammer M, et al (2010) Subgenual cingulate and visual cortex responses to sad faces predict clinical outcome during antidepressant treatment for depression. J Affect Disord 120: 120–125. 10.1016/j.jad.2009.04.031 19539998

[42] Whalen PJ, Johnstone T, Somerville LH, Nitschke JB, Polis S, et al (2008) A functional magnetic resonance imaging predictor of treatment response to venlafaxine in generalized anxiety disorder. Biol Psychiatry 63: 858–863. 17964548 10.1016/j.biopsych.2007.08.019 PMC2654286

[43] Goldstein AN, Greer SM, Saletin JM, Harvey AG, Nitschke JB, et al (2013) Tired and apprehensive: anxiety amplifies the impact of sleep loss on aversive brain anticipation. J Neurosci 33: 10607–10615. 10.1523/JNEUROSCI.5578-12.2013 23804084 PMC3693050

[44] Barlow DH (2001) Anxiety and its disorders. New York, NY: The Guilford Press.

[45] Grupe DW, Nitschke JB (2013) Uncertainty and anticipation in anxiety: an integrated neurobiological and psychological perspective. Nat Rev Neurosci 14: 488–501. 10.1038/nrn3524 23783199 PMC4276319

[46] Hamilton M (1959) The assessment of anxiety states by rating. Br J Med Psychol 32: 50–55. 13638508 10.1111/j.2044-8341.1959.tb00467.x

[47] Hamilton M (1960) A rating scale for depression. J Neurol Neurosurg Psychiatry 23: 56–62. 14399272 10.1136/jnnp.23.1.56 PMC495331

[48] Meyer TJ, Miller ML, Metzger RL, Borkovec TD (1990) Development and validation of the Penn State Worry Questionnaire. Behav Res Ther 28: 487–495. 2076086 10.1016/0005-7967(90)90135-6

[49] Buhr K, Dugas M.J. (2002) The intolerance of uncertainty scale: psychometric properties of the English version. Behaviour Research and Therapy 40: 931–945. 12186356 10.1016/s0005-7967(01)00092-4

[50] Lang PJ, Bradley B, Cuthbert B (1999) International affective picture system: Technical manual and affective ratings. Gainesville, FL: University of Florida.

[51] Wendland JR, Martin BJ, Kruse MR, Lesch KP, Murphy DL (2006) Simultaneous genotyping of four functional loci of human SLC6A4, with a reappraisal of 5-HTTLPR and rs25531. Mol Psychiatry 11: 224–226. 16402131 10.1038/sj.mp.4001789

[52] Jezzard P, Clare S (1999) Sources of distortion in functional MRI data. Hum Brain Mapp 8: 80–85. 10524596 10.1002/(SICI)1097-0193(1999)8:2/3<80::AID-HBM2>3.0.CO;2-C PMC6873315

[53] Lancaster JL, Woldorff MG, Parsons LM, Liotti M, Freitas CS, et al (2000) Automated Talairach atlas labels for functional brain mapping. Hum Brain Mapp 10: 120–131. 10912591 10.1002/1097-0193(200007)10:3<120::AID-HBM30>3.0.CO;2-8 PMC6871915

[54] Hedges L, Olkin I. (1985) Statistical methods for meta-analysis. New York, NY: Academic Press.

[55] Etkin A, Prater K, Schatzberg A, Menon V, Greicius M (2009) Disrupted amygdalar subregion functional connectivity and evidence of a compensatory network in generalized anxiety disorder. Archives of general psychiatry 66: 1361 10.1001/archgenpsychiatry.2009.104 19996041 PMC12553334

[56] Eickhoff S, Heim S, Zilles K, Amunts K (2006) Testing anatomically specified hypotheses in functional imaging using cytoarchitectonic maps. Neuroimage 32: 570–582. 16781166 10.1016/j.neuroimage.2006.04.204

[57] Bauernfeind AL, de Sousa AA, Avasthi T, Dobson SD, Raghanti MA, et al (2013) A volumetric comparison of the insular cortex and its subregions in primates. J Hum Evol 64: 263–279. 23466178 10.1016/j.jhevol.2012.12.003 PMC3756831

[58] Somerville LH, Wagner DD, Wig GS, Moran JM, Whalen PJ, et al (2013) Interactions between transient and sustained neural signals support the generation and regulation of anxious emotion. Cereb Cortex 23: 49–60. 10.1093/cercor/bhr373 22250290 PMC3513951

[59] Drabant EM, Ramel W, Edge MD, Hyde LW, Kuo JR, et al (2012) Neural mechanisms underlying 5-HTTLPR-related sensitivity to acute stress. Am J Psychiatry 169: 397–405. 10.1176/appi.ajp.2011.10111699 22362395 PMC3761065

[60] Carleton RN, Mulvogue MK, Thibodeau MA, McCabe RE, Antony MM, et al (2012) Increasingly certain about uncertainty: Intolerance of uncertainty across anxiety and depression. J Anxiety Disord 26: 468–479. 10.1016/j.janxdis.2012.01.011 22366534

[61] Mueller A, Armbruster D, Moser DA, Canli T, Lesch KP, et al (2011) Interaction of serotonin transporter gene-linked polymorphic region and stressful life events predicts cortisol stress response. Neuropsychopharmacology 36: 1332–1339. 10.1038/npp.2011.11 21368747 PMC3096802

[62] Williams LM, Gatt JM, Schofield PR, Olivieri G, Peduto A, et al (2009) ‘Negativity bias’ in risk for depression and anxiety: brain-body fear circuitry correlates, 5-HTT-LPR and early life stress. Neuroimage 47: 804–814. 10.1016/j.neuroimage.2009.05.009 19446647

[63] Button KS, Ioannidis JP, Mokrysz C, Nosek BA, Flint J, et al (2013) Power failure: why small sample size undermines the reliability of neuroscience. Nat Rev Neurosci 14: 365–376. 10.1038/nrn3475 23571845

[64] Meyer-Lindenberg A, Weinberger DR (2006) Intermediate phenotypes and genetic mechanisms of psychiatric disorders. Nat Rev Neurosci 7: 818–827. 16988657 10.1038/nrn1993

[65] Kalin NH, Shelton SE, Fox AS, Rogers J, Oakes TR, et al (2008) The serotonin transporter genotype is associated with intermediate brain phenotypes that depend on the context of eliciting stressor. Mol Psychiatry 13: 1021–1027. 10.1038/mp.2008.37 18414408 PMC2785009

[66] Monk CS, Klein RG, Telzer EH, Schroth EA, Mannuzza S, et al (2008) Amygdala and nucleus accumbens activation to emotional facial expressions in children and adolescents at risk for major depression. Am J Psychiatry 165: 90–98. 17986682 10.1176/appi.ajp.2007.06111917

[67] Stein MB, Simmons AN, Feinstein JS, Paulus MP (2007) Increased amygdala and insula activation during emotion processing in anxiety-prone subjects. Am J Psychiatry 164: 318–327. 17267796 10.1176/ajp.2007.164.2.318

