# Supplementary figures and images for: Affective Neural Responses Modulated by Serotonin Transporter Genotype in Clinical Anxiety and Depression
Source: PLoS One. 2015 Feb 12;10(2):e0115820. doi: 10.1371/journal.pone.0115820 (PMC4344476; doi:10.1371/journal.pone.0115820)

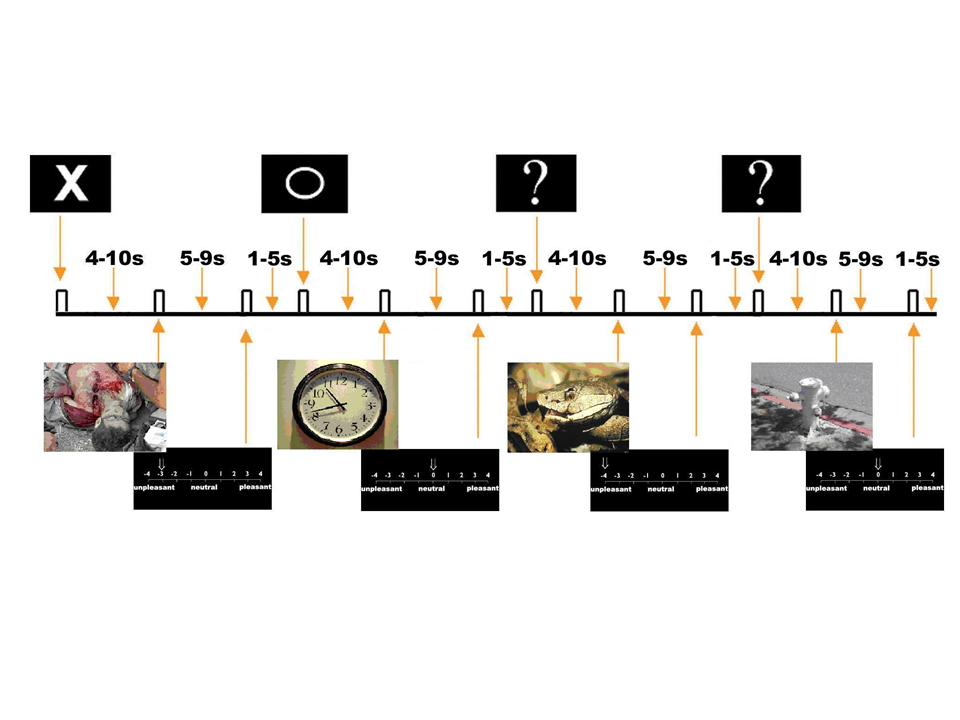

Supplement: S1 Fig — The experimental paradigm included three types of warning cues (X, O, ?). The ‘X’ cue indicated that an aversive picture would be presented; the ‘O’ cue indicated that a neutral picture would be presented; the ‘?’ cue indicated an equal probability of either an aversive or neutral picture. Warning cues were presented for two seconds. Pictures from the International Affective Picture System (IAPS) were presented for one second. Ratings of pictures or mood (50% of each, counterbalanced by picture valence) were presented for 5 seconds following each picture. ISIs between cue and picture ranged from 4–10 seconds, between picture and rating scale varied between 5–9 seconds, and between rating scale and subsequent warning cue ranged between 1–5 seconds. Aversive and neutral pictures were equated for luminance, and men and women viewed slightly different aversive picture sets based on normative ratings (36). Pictures shown below are not from IAPS and are for illustration purposes only. In addition to the 114 cue-picture trials described in the text, there were 14 catch trials for which no picture was presented and 12 catch trials for which a picture was presented with no warning cue (counterbalanced by valence). The fMRI data from these catch trials were not analyzed for this report. On the catch trials without a picture, a rating scale for anticipatory anxiety was presented for 5 s. Using a response box during the fMRI experiment, participants provided mood or picture ratings after the presentation of all pictures on an 11-point rating scale (-5 for “unpleasant,” 0 for “neutral,” 5 for “pleasant”) and anticipatory anxiety ratings after the cues on catch trials without a picture (0 for “Not at all,” 4 for “Moderately,” 8 for “Extremely”). Approximately one week before the fMRI experimental session, all subjects were positioned in a mock scanner, including head coil, goggles, and response box. After being instructed about all cue-picture pairings, subjects viewed an abbrevi [file pone.0115820.s001.tif]
